# Screening for depressive symptoms in adolescents at school: New validity evidences on the short form of the Reynolds Depression Scale

**DOI:** 10.1371/journal.pone.0170950

**Published:** 2017-02-21

**Authors:** Javier Ortuño-Sierra, Rebeca Aritio-Solana, Félix Inchausti, Edurne Chocarro de Luis, Beatriz Lucas Molina, Alicia Pérez de Albéniz, Eduardo Fonseca-Pedrero

**Affiliations:** 1 Department of Psychology, Universidad Loyola Andalucía, Sevilla, Spain; 2 Department of Educational Sciences, University of La Rioja, Logroño, Spain; 3 Navarre’s Health System, Pamplona, Spain; 4 Department of Psychology, University of Valencia, Valencia, Spain; Public Library of Science, FRANCE

## Abstract

The main purpose of the present study was to assess the depressive symptomatology and to gather new validity evidences of the Reynolds Depression Scale-Short form (RADS-SF) in a representative sample of youths. The sample consisted of 2914 adolescents with a mean age of 15.85 years (*SD* = 1.68). We calculated the descriptive statistics and internal consistency of the RADS-SF scores. Also, confirmatory factor analyses (CFAs) at the item level and successive multigroup CFAs to test measurement invariance, were conducted. Latent mean differences across gender and educational level groups were estimated, and finally, we studied the sources of validity evidences with other external variables. The level of internal consistency of the RADS-SF Total score by means of Ordinal alpha was .89. Results from CFAs showed that the one-dimensional model displayed appropriate goodness of-fit indices with CFI value over .95, and RMSEA value under .08. In addition, the results support the strong measurement invariance of the RADS-SF scores across gender and age. When latent means were compared, statistically significant differences were found by gender and age. Females scored 0.347 over than males in Depression latent variable, whereas older adolescents scored 0.111 higher than the younger group. In addition, the RADS-SF score was associated with the RADS scores. The results suggest that the RADS-SF could be used as an efficient screening test to assess self-reported depressive symptoms in adolescents from the general population.

## Introduction

Adolescence is a particularly important developmental stage for socio-emotional development, but it is also marked by the emergence of mental health problems like stress and anxiety, psychotic spectrum disorders or depressive disorders among others [1,2]. Specifically, major depressive disorder (MDD) is considered one of the leading causes of disability among young people aged 10–24 years [3]. MDD in children and adolescents affects different spheres and has been associated with poor academic performance, substance abuse, attempted and completed suicide, and an increased risk of suffering depression during adulthood [4,5]. Overall, the childhood and adolescent prevalence rates fall within the range of 3–8% [6,7]. In addition, between 20% and 50% of adolescents exhibit subclinical levels of depression [8,9]. Thus, efforts are currently aimed at early detection and treatment of children and adolescents with depressive symptoms that may delay or prevent the onset of the clinical outcome. With this regard, we need brief, well-validated, and psychometrically sound assessment tools.

There are a number of instruments with adequate psychometric properties in the literature, such as the Children´s Depression Scale (CDS) [10], the Children´s Depression Inventory (CDI) [11] or the Reynolds Adolescent Depression Scale (RADS) [12,13]. In particular, the RADS is a self-report measure composed of 30 items designed to assess the severity of depressive symptoms in adolescents, both in school and clinical settings [12,13]. The second version of the RADS (RADS-2), has shown adequate psychometric properties, including internal consistency measured by means of Cronbach’s alpha that ranged from 0.91 to 0.94 for the Total Depression Score across gender and groups (12). Also, the RADS-2 distinguished between clinical and non-clinical adolescents with a sensitivity of 92% and specificity of 84%. Evidences of correlations with other depressive symptoms, as well as suicidal ideations and anxiety, were also gathered (12). More recently, and with the aim to reduce completion time and make it more accessible to administer, the RADS-2 was shortened. The new short-form version of the instrument version (RADS-SF) is a 10-item unidimensional scale [14] that covers the same four subdimensions of the original RADS: dysphoric mood, anhedonia (negative affect), negative self-evaluation, and somatic complains, but that is supposed to be essentially unidimensional [14]. The RADS-SF was found to be effective in identifying self-report depressive symptoms in adolescents, showing similar psychometric properties as the original version (14).

Apart from the standardized manual of the RADS [14] that provided data about the reliability of the scores as well as different evidences of factorial validity, there are only two studies that have analysed the psychometric properties of the RADS-SF [14–16]. As an example, Szabo et al. [15], in a sample of 8,692 school students in New Zealand, revealed that the factorial structure was unidimensional, although better goodness-of-fit indices were found after adding some correlated errors. They also confirmed configural, scalar, and metric equivalence of the scores across gender, age, and ethnic groups, as well as strong associations with external variables like suicidal ideation, anxiety, and well-being. Reliability of the scores, estimated by means of Cronbach’s alpha was also adequate with a value of .88. It should be noted that the use of Ordinal alpha, more adequate when data are considered categorical, may have been more adequate in order to measure the reliability of the scores.

Similarly, a previous study, conducted also in New Zealand [16], with more than 9,500 secondary school students, found that the RADS-SF scores had adequate psychometric properties. In particular, reliability of the scores by means of Cronbach’s alpha was over .88, the factor structure yielded a good fit to the data, and the RADS-SF scores showed a strong correlation with the RADS. The RADS-SF was found to be invariant across gender and ethnic groups, although only configural invariance was tested. Another study [17] revealed the association between depression and suicidal ideation using the RADS-SF.

To date, and although the RADS-SF seems to be an appropriate measurement instrument to assess this construct, only few studies could be identified using this short form of the measure [15,16,18,19]. For instance, studies about the psychometric properties of the RADS-SF scores, including the analysis of MI, have not been yet conducted in Europe. Thus, there is a lack of studies addressing the reliability and new sources of validity evidence of the instrument around the world in order to be considered a useful tool for early detection of depressive symptoms (clinical and subclinical) in children and adolescents.

Taking everything into account the lack of previous research, the reliability of the scores, as well as the internal structure of the RADS-SF, are questions that still remain unclear. In addition, and although the RADS-SF is being used in adolescent populations, only few studies have analyzed the measurement equivalence of the instrument across gender and age. If MI does not hold, inferences and interpretations drawn from the data may be erroneous or unfounded [20,21]. Also, new data about the manifestation of depressive symptoms in adolescents with this measure is still needed and new sources of validity evidence with other measures are valuable.

Within this framework, the main objective of the current study was to assess the self-reported depressive symptoms and to analyze the psychometric properties of the RADS-SF scores in a large sample of non-clinical adolescents. Derived from this main goal are the following objectives: a) to estimate the reliability of the RADS-SF scores by means of the Ordinal alpha; b) to study the internal structure of the RADS-SF using CFAs; c) to study the measurement equivalence of the unidimensional model of the RADS-SF across gender and age; d) to compare latent mean scores across gender and age; and e) to analyze the sources of validity evidence with the RADS. In line, with previous literature, we hypothesized that the one-factor model of the RADS-SF would have adequate goodness-of-fit indices. In addition, we further hypothesized that the RADS-SF would be equivalent across gender and age. Finally, we expected that the reliability estimation of the RADS-SF scores would be adequate across groups.

## Method

### Participants

Stratified random cluster sampling was conducted at the classroom level, in an approximate population of 37,000 students selected from the Principality of Asturias and La Rioja, two regions located in northern Spain. The students belonged to different public and concerted Educational Centers of Compulsory Secondary Education and Vocational Training, as well as to different socio-economic levels. The layers were created as a function of the geographical zone and the educational stage. Partial data of this study have been published elsewhere [22]. There were 3052 students in the initial sample, although some participants were excluded due to their high scores on the Infrequency scale (more than three points) (*n* = 48), being older than 19 years of age (*n* = 20), not completing demographical data (e.g., gender and age) (*n* = 18) or not completing all the administered self-reports (*n* = 52). Thus, the final sample was composed of 2914 students, 1287 males (44.3%) from 41 centers and 95 classrooms. The mean age was 15.90 years (*SD* = 1.44), with an age range between 13 and 19 years. The distribution by age was: 13 years-old (n = 65), 14 year-olds (*n* = 425), 15 year-olds (*n* = 775), 16 year-olds (*n* = 712), 17 year-olds (*n* = 499), 18 year-olds (*n* = 285), and 19 year-olds (*n* = 153).

### Instruments

The Reynolds Adolescent Depression Scale (RADS) [13] is used to assess the severity of depressive symptomatology in adolescents from 11 to 20 years of age. It is composed of 30 items in a Likert response format with 4 options (1 = almost never, 2 = hardly ever, 3 = sometimes, 4 = most of the time). The total scores range from 30 to 120, with the cut-off score for determining the severity of the depressive symptomatology set at 77 points [13]. Reynolds [12] proposed four scales: Anhedonia/ Negative Affect, Somatic Complaints, Negative Self-Evaluation and Dysphoric Mood. The RADS has been extensively employed in a variety of topics, samples and nationalities presenting adequate psychometric properties [23–27]. The Spanish version, was used for the present study [28–30].

The RADS-SF consists of 10 critical items (1, 3, 6, 7, 14, 19, 20, 22, 28, and 30) from the original RADS that enables discriminating between depressed and non-depressed adolescents. In the present study, these 10 items were selected from the RADS. The RADS-SF, and according to the original version, has a 4-options Likert response format.Total scores range from 10 to 40. Distribution of the RADS-SF items by subdimensions is dysphoric mood (Items 3, 6, 7), anhedonia (Item 1), negative self-evaluation (Items 14, 19, 20, 30), and somatic complaints (Items 22, 28). Adequate psychometric properties have been found in previous studies [15,16].

The Oviedo Infrequency Scale (INF-OV) [31] was administered to the participants to detect those who responded in a random, pseudorandom or dishonest manner. The INF-OV instrument is a self-report composed of 12 items in a 5-point Likert- scale format (1 = completely disagree; 5 = completely agree) which has been developed following guidelines for test construction [32]. Items of the INF-OV included questions like for instance: “The distance between Madrid and New York is higher that the distance between Madrid and Barcelona”. Students with more than three incorrect responses on the INF-OV scale were eliminated from the sample. For this study, a total of 48 participants were excluded based on their responses to the INF-OV scale.

### Procedure

Contact with the principals of Compulsory Secondary Education and Vocational Training centers was made by letter or telephone. The administration of the questionnaires was conducted in a collective manner in groups of 15–25 participants, in a classroom within the school timetable. Students were reminded that their participation was voluntary and that their responses would be kept confidential. Written informed consent to participate in the study was obtained from the adolescents. For participants under 18, parents were asked to provide written informed consent in order to allow their children to participate in the study. Participants did not receive any type of incentive for their participation in the study. The administration took place under the supervision of the researchers. The study was approved by the Research and Ethics Committee at University of La Rioja and University of Oviedo.

### Data analyses

First, we calculated the descriptive statistics and internal consistency of the RADS-SF scores. Ordinal alpha coefficient for Likert data was calculated as a measure of the reliability of the scores [33]. Ordinal alpha is conceptually equivalent to Cronbach’s alpha and it is more adequate for dichotomous and ordinal data. The critical difference between the two is that Ordinal alpha is based on polychoric correlation matrix, rather than Pearson matrix.

Second, we conducted several CFAs at the item level: 1) In the first step, we tested the hypothesis of the RADS-SF being one-dimensional; 2) In addition, as proposed by Szabo et al., (15) we tested the one-dimensional including the error covariance of items 19–20, as both intend to measure the same symptom, items 3–6, and 3–7 as they all belong to the dysphoric mood subdimension. Due to the ordinal nature of the data, we used the Weighted Least Squares Means and Variance adjusted (WLSMV) estimator, and the polychoric correlation matrix.The following goodness-of-fit indices were used: Chi-square (χ^2^), Comparative Fit Index (CFI), Tucker-Lewis Index (TLI), Root Mean Square Error of Approximation (RMSEA) (and 90% confidence interval), and Weighted Root Mean Square Residual (WRMR). The CFI values greater than .95 are preferred and values close to .90 are considered acceptable, while the RMSEA values should be under .08 for a reasonable fit, and under .05 for a good fit [34,35]. For the WRMR values < 1.0 have been suggested as indicative of adequate model fit [36]. Within the multilevel CFA framework, and due to the hierarchical structure of the data (participants nested in clasrooms), we also studied the intraclass correlation coefficients (ICCs) of the observed variables (items of the RADS-SF) attending to the class level. The ICC assesses the level of variance in the observed variable that is attributable to membership in its cluster. ICC values range from 0.0 to 1.0.

Third, in order to test MI, successive multigroup CFAs were conducted [37]. Basically, a hierarchical set of steps are followed when MI is tested, typically starting with the determination of a well-fitting multigroup baseline model and continuing with the establishment of successive equivalence constraints in the model parameters across groups. The analysed dimensional models can be seen as nested models to which constraints are progressively added. Using Delta parameterization in Mplus, two steps on measuring invariance need to be considered: configural and strong invariance models [38].

Due to the limitations of the Δχ^2^ regarding its sensitivity to sample size, Cheung and Rensvold [39] proposed a more practical criterion, the change in CFI (ΔCFI), to determine if nested models are practically equivalent. In this study, when ΔCFI is greater than .01 between two nested models, the more constrained model is rejected since the additional constraints have produced a practically worse fit. However, if the change in CFI is less than or equal to .01, it is considered that all specified equal constraints are tenable, and therefore, it is possible to continue with the next step in the analysis of MI.

Fourth, latent mean differences across gender and educational level groups were estimated, fixing the latent mean values to zero in the male and in the high school students groups. For comparisons among groups in the latent means, statistical significance was based on the z statistic. The group in which the latent mean was fixed to zero was considered as the reference group. Finally, we studied the sources of validity evidences with other external variables. We analysed the correlation between the RADS-SF and the RADS by means of Pearson correlations. SPSS 15.0 [40], Factor 9.2 [41], and Mplus 7.0 [38] were used for data analyses.

## Results

### Descriptive statistics and internal consistency of the scores

Table 1 shows the descriptive statistics referring to the mean and standard deviation for the RADS-SF items by gender and age, and the total sample. The Ordinal alpha for the Total Score was .89, indicating good levels of reliability of the scores. According to gender, Ordinal alpha for gender was .87 for males and for .91 females. With regards to the age, Ordinal alpha was .88 for the younger group (13–15 years old) and .90 for the older group (16–19 years old). Moreover and as another indicator of the internal consistency of the scores, we calculated the average inter–item correlation (AIC) for the whole sample. The AIC was .45 (range .25 to .73)

**Table 1:** Descriptive statistics of the RADS-SF items.

	Male (*n* = 1287)		Female (*n* = 1627)		13–15 years (*n* = 1265)		16–19 years (*n* = 1649)		Total (*N* = 2914)	
Ordinal alpha	.89		.89		.88		.89		.90	
Items	Mean	*SD*	Mean	*SD*	Mean	*SD*	Mean	*SD*	Mean	*SD*
1	1.57	0.73	1.55	0.70	1.51	0.69	1.59	0.73	1.56	0.71
3	1.53	0.76	1.70	0.82	1.58	0.79	1.66	0.80	1.62	0.80
6	1.55	0.79	1.65	0.82	1.56	0.81	1.63	0.81	1.60	0.81
7	1.65	0.73	1.98	0.79	1.80	0.80	1.87	0.77	1.84	0.78
14	1.14	0.50	1.15	0.49	1.16	0.52	1.14	0.47	1.15	0.49
19	1.43	0.69	1.38	0.65	1.39	0.66	1.41	0.68	1.40	0.67
20	1.36	0.68	1.49	0.78	1.44	0.75	1.43	0.73	1.43	0.74
22	2.69	0.81	2.70	0.79	2.72	0.83	2.68	0.77	2.69	0.80
28	2.27	0.90	2.28	0.83	2.30	0.88	2.26	0.85	2.28	0.86
30	1.60	0.82	1.72	0.85	1.67	0.86	1.67	0.83	1.67	0.84

*Note*. *SD* = Standard Deviation.

### Internal structure of the RADS-SF: Confirmatory factor analysis

The goodness-of-fit indices for the RADS-SF models estimated by means of CFAs are presented in Table 2. As it can be seen, the one dimensional model showed a good fit to the data, with CFI values over .95, and RMSEA values under .08. We also tested a model with the inclusion of error correlation between items 19–20, 3–6 and 3–7 as suggested by Szabo et al. [15]. This resulted in an improvement of the goodness-of-fit indices. Nevertheless, due to the inherent problematic in the use of correlated errors [42], and the fact that the one-dimensional model displayed a good adjust we decided not to take into consideration the correlated errors. The standardized factor loadings for the one-dimensional model, attending to gender, age and the final sample, are shown in Table 3. All the factor loadings, for the RADS-SF were significant, ranging, in the total sample, between .35 (*Self-Deprecation*, *Low Self-Worth*) and .82 (*Anger*, *Irritability*). With regards to the multilevel CFA study, all the items showed ICC values lower than .10, indicating that hierarchical nature of the data did not have a significant effect in the dimensional structure of the RADS-SF. Thus, the amount of variance attributable to cluster membership was lower than 10%, suggesting that a multilevel analysis is not required.

**Table 2:** Goodness of fit indices for the hypothetical models tested and measurement invariance across gender and age.

Model	χ^2^	*df*	CFI	TLI	RMSEA (CI 90%)	WRMR	ΔCFI
1 factor	620.50	35	.95	.94	.07 (.07-.08)	2.27	
1 factor + CE*	513.89	32	.96	.95	.07 (.06-.07)	2.01	
*Measurement Invariance for the one factor model*							
**Gender**							
Male (*n* = 1287)	319.87	35	.95	.93	.08 (.07-.09)	1.62	
Female (*n* = 1627)	369.26	35	.96	.94	.08 (.07-.08)	1.73	
Configural invariance	638.74	73	.96	.95	.07 (.07-.08)	2.35	
Strong invariance	778.85	89	.95	.94	.07 (.07-.08)	2.75	-.01
**Age**							
13–15 years old (*n* = 1265)	293.53	35	.95	.94	.08 (.07-.09)	1.55	
16–19 years old (*n* = 1649)	353.01	35	.96	.94	.07 (.07-.08)	1.71	
Configural invariance	643.49	73	.95	.94	.07 (.07-.08)	2.35	
Strong invariance	692.02	89	.95	.95	.07 (.07-.08)	2.54	-.01

*Note*. CE = Correlated Errors; χ^2^ = Chi square; *df* = degrees of freedom; CFI = Comparative Fit Index; TLI = Tucker-Lewis Index; RMSEA = Root Mean Square Error of Approximation; CI = Confidence Interval; WRMR = Weighted Root Mean Square Residual; ΔCFI = Change in Comparative Fix Index.

*CE = 3 with 6; 3 with 7; 19 with 20.

**Table 3:** Standardized factor loadings for the configural final model.

	Male	Female	13–15 years	16–19 years	Whole Sample
Items	Loadings (R^2^)	Loadings (R^2^)	Loadings (R^2^)	Loadings (R^2^)	Loadings (R^2^)
1	.32 (.68)	.28 (.72)	.29 (.71)	.29 (.44)	.54 (.29)
3	.52 (.49)	.52 (.48)	.50 (.50)	.54 (.52)	.72 (.52)
6	.37 (.63)	.42 (.58)	40 (.60)	.40 (.18)	.64 (.40)
7	.54 (.46)	.53 (.47)	.54 (.46)	.53 (.47)	73 (.54)
14	.47 (.53)	.56 (.44)	.51 (.48)	.53 (.54)	.72 (.52)
19	.23 (.77)	.27 (.73)	.25 (.75)	.23 (.77)	.48 (.24)
20	.66 (.34)	.67 (.33)	.68 (.32)	.67 (.33)	.82 (.68)
22	.14 (.86)	.11 (.89)	.17 (.83)	.09 (.91)	.35 (.12)
28	.24 (.76).	.15 (.85)	.17 (.83)	.20 (.80)	.43 (.18)
30	.59 (.42)	.61 (.39)	.63 (.38)	.59 (.41)	.78 (.60)

*Note*. All standardized factorial loadings estimated were statistically significant (*p* < 0.01).

### Measurement invariance of the RADS-SF scores gender and age

Prior to the analysis of MI across gender and age, we tested whether the one-dimensional model showed a reasonable good fit to the data in each group separately. First, the MI of the RADS-SF across gender was tested. The configural invariance model in which no equality constraints were imposed showed an adequate fit to the data. Next, a strong invariance model was tested with the item thresholds and factor loadings constrained to be equal across groups. The results showed configural and strong MI by gender. Next, the MI of the RADS-SF across age was tested. To examine MI by age, the sample was divided into two subgroups (13–15 year-olds and 16–19 year-olds), according to the early and middle and final stages of the adolescence [43]. The ΔCFI between the constrained and the unconstrained model was under .01, indicating that the model of strong invariance was supported. Hence, the results support strong MI by gender and age.

### Latent mean scores across gender and age

For comparisons among groups in the latent means, statistical significance was based on the *z* statistic. The group in which the latent mean was fixed to zero was considered as the reference group. With regards to the gender, statistically significant differences were found. Specifically, females scored 0.347 units over than males in Depression (0.347; *p* < .01). The comparison across groups in latent means also revealed statistically significant differences according to age. Thus, the comparison across groups in latent means indicated that, on average, younger adolescents scored 0.111 units under older adolescents in Depression (0.111; *p* < .05).

### Sources of validity evidence with RADS scores

In order to test for convergent validity, Pearson correlation between the total score of the RADS and total score of the RADS-SF was calculated. Results showed a correlation of .91, indicating a high correlation between the two scales.

## Discussion

The main purpose of the present study was to study the psychometric properties of the RADS-SF [44] in a large sample of adolescents. We thus estimated the reliability of the scores, examined the internal structure and MI across gender and age of the RADS-SF scores, and gathered different sources of validity evidence with other measures. Results found in the study show that the RADS-SF scores has adequate psychometric properties in this sample and, therefore, that it is a useful instrument that could be used to evaluate self-reported depressive symptoms in non-clinical populations of adolescents.

The reliability of the scores, estimated with Ordinal alpha, was adequate, with a value of .88. To the best of our knowledge, no previous studies have estimated the reliability of the RADS-SF scores using the Ordinal alpha. These results are consistent with previous studies, indicating that the RADS-SF scores had adequate levels of internal consistency estimated with Cronbach’s alpha. Future studies should analyze the reliability of the RADS-SF scores through Ordinal alpha due to the ordinal nature of the variables.

Results of the CFAs showed that the one-dimensional model yielded adequate goodness-of-fit indices. Similar to the results found in this study, previous research suggested that the RADS-SF has a unidimensional structure [15,16,45]. In addition, and attending to the findings of Szabo et al. [15], the inclusion of different correlated errors was tested. The inclusion of the correlated errors produced an increase in some goodness-of- fit indices. However, we decline its use, due to the inherent problematic in the use of correlated errors [42] and the fact that the model only contained ten items, making the use of correlated errors even more controversial. It is worth noting, that we used the WLSMV estimator for our analysis due to the categorical nature of the data. A four Likert-format response scale, as it is the case of the RADS-SF, should be considered as categorical. Future studies could analyze the internal structure of the instruments considering data as categorical.

Also, the MI of the RADS-SF across gender and age (early adolescent and middle and late adolescent) was tested. The results supported the hypothesis of strong MI by gender and age in our sample. Thus, the RADS-SF showed factorial equivalence by gender and age. These results are somehow similar to other studies that have found total factorial equivalence of the RADS-SF scores across different variables (e.g., gender) [15,45]. For instance, Szabo et al [15] found metric MI in a large sample of New Zealand adolescents across different variables, including gender and age. The other study conducted with the analyzing the structural equivalence of the instrument only analyzed configural invariance across gender and age, so the results found in the present study contribute relevant information.

With this regard, more studies analyzing the MI of the RADS-SF, with data considered as ordinal are needed. It should be stressed, that if MI does not hold, the suggestion is that the validity of such scores should be questioned. As such, it is critical for MI conclusions to be based on statistically sound results. The comparability between different groups only makes sense if it can be guaranteed that participants interpret and understand the latent construct in a similar manner. Hence, from a psychometric point of view, the study of MI is a prerequisite for performing any group comparisons [21,37]. Therefore, if any difference in the latent mean score is found, we can be sure that such difference is a result of a true difference in the latent variable, and not a measurement artifact.

The comparison in the latent means across gender and age yielded statistically significant differences. Females obtained higher scores than males. As a function of age, adolescents between 16 and 19 years obtained higher scores compared to the younger group. Consistent with the previous literature, the expression of depressive symptomatology varies as a function of age and gender [12,25,30,46]. In general terms, the prevalence of depressive symptoms has an earlier onset in females than in males [47] and increases with age, being more frequent in adolescents than in children [48]. Using the raw scores of the RADS subscales or the RADS total score, we found that female adolescents obtained higher scores than males in depressive dimensions except in Anhedonia where males revealed higher scores and the older adolescents also obtained higher scores in comparison to the younger adolescents

Results from the analysis of the sources of convergent validity evidence yielded a significant high association, over .90, between the RADS-SF scores and the RADS. These results are consistent with previous studies analyzing criterion validity of the RADS-SF. For instance, Milfont et al. [16] revealed a correlation over .90 between the RADS-SF and the RADS, similarly to the results found in the present work.

The results found in this study should be interpreted in light of some possible limitations. First, the sample was composed exclusively of adolescents. It is well known that adolescence is a developmental period where a great variety of neuromaturational, social, emotional, and self-identity changes occur [49] and that may have an influence in the phenomenological expression of this construct. Second, in this study, information was gathered based solely on self-reports, for which, we consider that it would have been interesting to complete this information with a clinical interview or with a hetero-report administered to the participants’ parents. Third, evidence for convergent and discriminant validity were not further explored. With this regard, it might have been useful to include other related and unrelated measures of the severity of depressive symptoms. Finally, fourth, it is also important to note that the item response theory (IRT) modeling could have been used in order to contribute new psychometric approaches in order to check for instance differences between groups such as males and females in target items. Despite the noted limitations, and areas that would benefit of future research, the present study adds more information about different sources of validity and about the reliability of the questionnaire, and identified the fruitfulness of the RADS-SF in order to be used as a measure of depression in non-clinical populations of adolescents.

Future studies could replicate these findings in other samples. Moreover, future research on the MI across cultures in the self-reported version would enable the comparison of results between different countries, regions or cultures with the RADS-SF version.

## References

[1] Beddington J, Cooper CL, Field J, Goswami U, Huppert FA, Jenkins R, et al The mental wealth of nations. 2008;455:1057–1060. 10.1038/4551057a 18948946

[2] Ortuño J, Fonseca-Pedrero E, Paino M, Aritio-Solana R. Prevalencia de síntomas emocionales y comportamentales en adolescentes españoles [Prevalence of emotional and behavioural symptoms in spanish adolescents]. Revista de Psiquiatría y Salud Mental 2014;7:121–130. 10.1016/j.rpsm.2013.12.003 24530346

[3] Gore FM, Bloem PJ, Patton GC, Ferguson J, Joseph V, Coffey C, et al Global burden of disease in young people aged 10–24 years: a systematic analysis. Lancet 2011 6 18;377(9783):2093–2102. 10.1016/S0140-6736(11)60512-6 21652063

[4] Bae SM, Lee SA, Lee SH. Prediction by data mining, of suicide attempts in Korean adolescents: a national study. Neuropsychiatric Disorders Treatment 2015;11(2367):2375. 10.2147/NDT.S91111 PMC4577255 26396521

[5] Merikangas KR, He J, Burstein M, Swanson SA, Avenoli S, Cui L, et al Lifetime Prevalence of Mental Disorders in U.S. Adolescents: Results from the National Comorbidity Survey Replication-Adolescents Spplement (NCS-A). Journal of the American Academic of Child and Adolescence Psychiatry 2010;49(10):980–989. 10.1016/j.jaac.2010.05.017 PMC2946114 20855043

[6] Kessler RC, Avenevoli S, Costello EJ, Georgiades K, Green JG, Gruber MJ, et al Prevalence, persistence, and sociodemographic correlates of DSM-IV disorders in the National Comorbidity Survey Replication Adolescent Supplement. Archives of General Psychiatry 2012;69(4):372–380. 10.1001/archgenpsychiatry.2011.160 22147808 PMC3445020

[7] Riglin L, Thapar A, Shelton KH, Langley K, Frederickson N, Rice F. Profiling depression in childhood and adolescence: the role of conduct problems. Journal Child Psychology Psychiatry 2015. 10.1111/jcpp.12465 PMC5102656 26400027

[8] Kessler RC, Avenevoli S, Ries Merikangas K. Mood disorders in children and adolescents: an epidemiologic perspective. Biol Psychiatry 2001 6 15;49(12):1002–1014. 11430842 10.1016/s0006-3223(01)01129-5

[9] Petersen AC, Compas BE, Brooks-Gunn J, Stemmler M, Ey S, Grant KE. Depression in adolescence. Am Psychol 1993 2;48(2):155–168. 8442570 10.1037//0003-066x.48.2.155

[10] Lang M, Tisher M. Children's Depression Scale (CDS) manual (North American Edition). Palo Alto, CA: Consulting Psychologists Press; 1987.

[11] Kovacs M. Manual of the Children’s Depression Inventory. Toronto: Multi-Heath Systems, Inc; 1992.

[12] Reynolds WM. Reynolds Adolescent Depression Scale– 2nd Edition. Professional manual. Odessa: Psychological Assessment Resources, Inc; 2002.

[13] Reynolds WM. Reynolds Adolescent Depression Scale. Professional manual. Odessa: Psychological Assessment Resources, Inc; 1987.

[14] Reynolds WM. Reynolds adolescent depression scale (2nd ed., Short Form-RADS-2:SF). Odessa, FL: Psychological Assessment Resources; 2008.

[15] Szabo A, Milfont TL, Merry SN, Robinson EM, Crengle S, Ameratunga SN, et al Equivalence of the Short Form of the Reynolds Adolescent Depression Scale across Groups. Journal of Clinical Child and Adolescent Psychology 2014;43:592–600. 10.1080/15374416.2013.848770 24246041

[16] Milfont TL, Merry SN, Robinson EM, Denny SJ, Crengle S, Ameratunga SN. Evaluating the short form of the Reynolds adolescent depression scale in New Zealand adolescents. Australian and New Zealand Journal of Psychiatry 2008;42:950–954. 10.1080/00048670802415343 18941959

[17] Horwitz AG, Hill RM, King CA. Specific coping behaviors in relation to adolescent depression and suicidal ideation. J Adolesc 2011 10;34(5):1077–1085. 10.1016/j.adolescence.2010.10.004 21074841 PMC3319342

[18] Crengle S, Robinson EM, Ameratunga SN, Clark T, Raphael D. Ethnic discrimination prevalence and associations with health outcomes: Data from a nationally representative cross-sectional survey of secondary school students in New Zealand. BMC Public Health 2012;12:45–56. 10.1186/1471-2458-12-45 22257643 PMC3315751

[19] Lucassen MFG, Merry SN, Robinson EM, Denny S, Clark T, Ameratunga S, et al Sexual attraction, depression, self-harm, suicidality and help-seeking behaviour in New Zealand secondary school students. Australian and New Zealand Journal of Psychiatry 2011;45:376–383. 10.3109/00048674.2011.559635 21361850

[20] Byrne B. Structural Equation Modeling with Mplus: Basic Concepts, Applications, and Programming. New York: Routledge Taylor & Francis Group; 2012.

[21] Meredith W. Measurement invariance, factor analysis and factorial invariance. Psychometrika 1993;58:525–543.

[22] Fonseca-Pedrero E, Wells C, Paino M, Lemos-Giráldez S, Villazón-García U, Sierra-Baigrie S, et al Measurement invariance of the Reynolds Depression Adolescent Scale across gender and age. International Journal of Testing, 2010;10:133–148.

[23] Reynolds WM. Children and adolescents: Clinical formulations and treatment In: Ollendick TH, editor. Comprehensive clinical psychology New York: Pergamon Press; 1998 p. 419–461.

[24] Reynolds WM, Mazza JJ. Reliability and validity of the Reynolds Adolescent Depression Scale with young adolescents. Journal of School Psychology. 1998;36:295–312.

[25] Walker L, Merry S, Watson PD, Robinson E, Crengle S, Schaaf D. The Reynolds Adolescent Depression Scale in New Zealand adolescents. Australian and New Zealand Journal of Psychiatry 2005;39:136–140. 10.1111/j.1440-1614.2005.01534.x 15701061

[26] Maharajh HD, Ali A, Konings M. Adolescent depression in Trinidad and Tobago. European Child & Adolescent Psychiatry 2006;15:30–37. 16514507 10.1007/s00787-006-0501-3

[27] Hyun M, Nam K, Kang HS, Reynolds WM. Reynolds Adolescent Depression Scale–Second Edition: initial validation of the Korean version. Journal of Advanced Nursing 2009;65:642–651. 10.1111/j.1365-2648.2008.04913.x 19222662

[28] Fonseca-Pedrero E, Wells C, Paino M, Lemos-Girádez S, Villazón-García U, Sierra S, et al Measurement invariance of the Reynolds Depression Adolescent Scale across gender and age. International Journal of Testing 2010;10:133–148.

[29] Fonseca-Pedrero E, Paino M, Lemos-Giraldez S, Muñiz J. Schizotypal traits and depressive symptoms in nonclinical adolescents. Comprehensive Psychiatry 2011;52(3):293–300. 10.1016/j.comppsych.2010.07.001 21497224

[30] Figueras-Masip A, Amador-Campos JA, Peró-Caballero M. Características psicométricas de la *Reynolds Adolescent Depression Scale* en población comunitaria y clínica [Psychometric characteristics of the RADS in community and clinical populations]. International Journal of Clinical and Health Psychology 2008;8:247–266.

[31] Fonseca-Pedrero E, Lemos-Giráldez S, Paino-Pineiro M, Villazón-García U, Muñiz J. Validation of the Schizotypal Personality Questionnaire-Brief Form in adolescents. Schizophrenia Research 2009;111:53–60. 10.1016/j.schres.2009.03.006 19342199

[32] Schmeiser CB, Welch C. Test development In: Brennan RL, editor. Educational Measurement Westport, CT: American Council on Education/Praeger; 2006 p. 307–353.

[33] Zumbo BM, Gadermann AM, Zeisser C. Ordinal versions of coefficients alpha and theta for Likert rating scales. Journal of Modern Applied Statistical Methods 2007;6:21–29.

[34] Brown TA. Confirmatory Factor Analysis for Applied Research. New York: Guilford Press; 2006.

[35] Hu LT, Bentler PM. Cut off criteria for fit indexes in covariance structure analysis: Conventional criteria versus new alternatives. Structural Equation Modeling 1999;6(1):1–55.

[36] Yu CY. Evaluating cutoff criteria of model fit indices for latent variable models with binary and continuous outcomes. Los Angeles: University of California, Doctoral dissertation; 2002.

[37] Byrne BM. Testing for multigroup equivalence of a measuring instrument: A walk through the process. Psicothema 2008;20:872–882. 18940097

[38] Muthén LK, Muthén BO. Mplus User's Guide. Seventh Edition Los Angeles: Muthén & Muthén; 2012.

[39] Cheung GW, Rensvold RB. Evaluating goodness-of-fit indexes for testing measurement invariance. Structural Equation Modeling 2002;9:233–255.

[40] Statistical Package for the Social Sciences. SPSS Base 15.0 User's Guide. 2006.

[41] Lorenzo-Seva U, Ferrando FJ. Factor: A computer program to fit the exploratory factor analysis model. 2006;38:88–91. 16817517 10.3758/bf03192753

[42] Heene M, Hilbert S, Freudenthaler HH, BÃ¼hner M. Sensitivity of SEM Fit Indexes With Respect to Violations of Uncorrelated Errors. Structural Equation Modeling 2012;19:36–50.

[43] Salmera-Aro K. Stages of Adolescence In: Bradford Brown B, Prinstein MJ, editors. Encyclopedia of Adolescence Oxford: Elsevier; 2011 p. 360–368.

[44] Reynolds WM. Reynolds adolescent depression scale (2nd ed., Short Form-RADS-2:SF). Odessa, FL: Psychological Assessment Resources; 2008.

[45] Milfont TL, Fisher R. Testing measurement invariance across groups: applications for cross-cultural research. International Journal of Psychological Research 2010;3:111–121.

[46] Carle AC, Millsap RE, Cole DA. Measurement bias across gender on the Children's Depression Inventory. Evidence for invariance from two latent variable models. Educational and Psychological Measurement 2008;68:281–303.

[47] Angold A, Erkanli A, Silberg J, Eaves L, Costello EJ. Depression scale scores in 8-17-year-olds: effects of age and gender. Journal Child and Psychology Psychiatry 2002;43:1052–1063. 10.1111/1469-7610.00232 12455926

[48] Costello EJ, Mustillo S, Erkanli A, Keeler G, Angold A. Prevalence and development of psychiatric disorders in childhood and adolescence. Archives of General Psychiatry 2003;60:837–844. 10.1001/archpsyc.60.8.837 12912767

[49] Kays JL, Hurley RA, Taber KH. The dynamic brain: neuroplasticity and mental health. Journal of Neurosychiatry and Clinical Neurosciences 2012;24:118–124. 10.1176/appi.neuropsych.24.1.118 22772660

